# Volatilome is Inflammasome- and Lipidome-dependent in Ischemic Heart Disease

**DOI:** 10.2174/011573403X302934240715113647

**Published:** 2024-07-19

**Authors:** Basheer Abdullah Marzoog

**Affiliations:** 1 World-Class Research Center «Digital Biodesign and Personalized Healthcare», I.M. Sechenov First Moscow State Medical University (Sechenov University), 119991 Moscow, Russia

**Keywords:** Lipidome, metabolome, VOCs, ischemic heart disease, inflammasome, coronary artery disease

## Abstract

Ischemic heart disease (IHD) is a pathology of global interest because it is widespread and has high morbidity and mortality. IHD pathophysiology involves local and systemic changes, including lipidomic, proteomic, and inflammasome changes in serum plasma. The modulation in these metabolites is viable in the pre-IHD, during the IHD period, and after management of IHD in all forms, including lifestyle changes and pharmacological and surgical interventions. Therefore, these biochemical markers (metabolite changes; lipidome, inflammasome, proteome) can be used for early prevention, treatment strategy, assessment of the patient's response to the treatment, diagnosis, and determination of prognosis. Lipidomic changes are associated with the severity of inflammation and disorder in the lipidome component, and correlation is related to disturbance of inflammasome components. Main inflammasome biomarkers that are associated with coronary artery disease progression include IL‐1*β*, Nucleotide-binding oligomerization domain-like receptor family pyrin domain containing 3 (NLRP3), and caspase‐1. Meanwhile, the main lipidome biomarkers related to coronary artery disease development involve plasmalogen lipids, lysophosphatidylethanolamine (LPE), and phosphatidylethanolamine (PE). The hypothesis of this paper is that the changes in the volatile organic compounds associated with inflammasome and lipidome changes in patients with coronary artery disease are various and depend on the severity and risk factor for death from cardiovascular disease in the time span of 10 years. In this paper, we explore the potential origin and pathway in which the lipidome and or inflammasome molecules could be excreted in the exhaled air in the form of volatile organic compounds (VOCs).

## 1 INTRODUCTION

Ischemic heart disease (IHD) is the pathology of the century in terms of mortality, morbidity, and costs [1]. Understanding the mechanism of the development of cardiovascular disease and the related systemic changes is a challenging issue. The proper evaluation of the molecular biopathophysiology is the cornerstone for the prevention and early diagnosis and thereafter, timely treatment of this disease.

The changes in the breath analysis in patients with ischemic heart disease are associated with the systemic endogenous changes that occur in these patients. Therefore, the development of a systemic strategy for analyzing the changes in the organism is essential to accurately evaluate the endogenous sources of the volatile organic compounds (VOCs) in the exhaled breath analysis. One of the well-studied changes in patients with ischemic heart disease are the lipidome and inflammasome changes.

The hypothesis that stands behind ischemic heart disease is atherosclerosis formation. The main pathological issues that occur in the development of atherosclerosis are dyslipidemia, high shear stress due to arterial hypertension, and chronic mild inflammation. Examination of the changes in the level of the lipidome and inflammasome can be used as a potential tool for early detection of cardiovascular disease or risk evaluation for the development of cardiovascular disease, including ischemic heart disease. Additionally, changes in the lipidome and inflammasome can be correlated, and a regression model can be built to identify if the changes in the exhaled breath analysis are correlated or not with the lipidome and inflammasome levels.

Coronary artery disease development is primarily due to atherosclerosis. According to a recent study, atherosclerosis development is inversely associated with the nitric oxide level in the exhaled breath [2]. This indicates a potential application of nitric oxide for the determination of coronary artery disease severity by detecting the size of the atherosclerosis and further the stenosis of the coronary arteries [2]. However, challenges emerge during the localization of atherosclerosis, such as peripheral artery disease in the extremities or the carotid arteries, as well as the large vessels [2]. These changes in the exhaled nitric oxide are inversely associated with triglyceride levels and glycosylated hemoglobin as well as plasma glucose levels [2].

Inflammation and dyslipidemia are two components strongly related to each other and directly involved in the development of coronary artery disease (CAD). Several biomarkers of inflammation have been seen to be related to CAD. Simultaneously, an elevation in some lipid biomarkers has been seen in CAD victims. Recent papers suggested that using the lipidome biomarkers as a novel pathway for evaluating the risk of developing cardiovascular disease can be of clinical value [3–6]. Additionally, a recent paper applied machine learning-based approaches to evaluate cardiovascular risk by analyzing the results of the lipidome biomarkers [3].

This paper provides an update on VOCs as a non-invasive method that can hold the key to detecting early metabolic pathway changes in ischemic heart disease development.

## 2 INFLAMMASOME CHANGES IN CORONARY ARTERY DISEASE

The disbalance between anti-inflammatory and pro-inflammatory substances characterizes CAD. The inflammasome is a complex of inflammation biomarkers that appears in the blood circulation after infection or tissue damage [7]. The inflammasome proteins complex is activated in two stages and involves the activation of the interleukins-1β and -18 [8]. Inflammasome is a wide term used to describe the inflammation biomarkers [7].

One of the well-studied proinflammatory agents is IL‐1β, which is produced after cholesterol crystal phagocytosis by phagocytes and loss of the lysosomal membrane integrity [9]. Suppression of the pro‐IL‐1*β* and NLRP3 and activation of caspase‐1 are potential methods for inflammasome downregulation [9].

A randomized, double-blind trial demonstrated that inhibition of the IL‐1β by 150 mg of canakinumab every 3 months significantly reduced the primary efficacy endpoint of nonfatal myocardial infarction, nonfatal stroke, or cardiovascular death in patients with previous myocardial infarction and a high-sensitivity C-reactive protein level of 2 mg or more per liter [10]. However, the reduction in the incidence rate was not associated with improvement in the lipid profile. This demonstrates the role of inflammasome in the pathogenesis and development of cardiovascular disease.

It is critical to mention the factors of coronary artery disease development, including endothelial cell dysfunction, lipid profile changes, inflammation, chronic shear stress, and hypertension [8, 11-17]. These factors collectively interact with each other to give the end picture of cardiovascular disease.

Interestingly, a recent *in vitro* study demonstrated that splenic monocytes play a critical role in the progression of the ischemia in coronary artery disease through the upregulation of the splenic NLRP3 inflammasome, particularly CD11b^+^ and LY6G^−^ splenocytes [18]. In addition, the knockdown of the NLRP3 by CY09 (especially micro molecule that can inhibit the NLRP3 in splenic monocytes) or adoptive transferring of splenic monocytes with NLRP3^-/-^ to a mouse with splenectomy was associated with a dramatical reduction or limitation in the infarcted/ischemic zone [18]. Furthermore, the splenic NLRP3 inflammasome is activated through the myocardial cell-free DNA (cfDNA) and especially through the mitochondrial cell-free DNA (mt-cfDNA) [18]. The inhibition of the mt-cfDNA by the Toll-like receptor 9 (TLR9) inhibitor is associated with the reduction of the infarcted/ischemic zone [18]. Therefore, targeting the NLRP3 inflammasome is a potential therapeutic targeting in terms of reduction and early prevention of the progression of the myocardial ischemic zone in patients with stable coronary artery disease.

According to a foundational study related to the inflammasome and coronary artery disease development, NLRP3 inflammasomes have been shown to be activated by the cholesterol crystals and are associated with atherosclerosis emergence [19]. Additionally, inflammasomes have a key role in cardiac fibroblast homeostasis regulation, which is strictly related to the modification of the ischemic reperfusion injury [20].

**Fig. (1):**
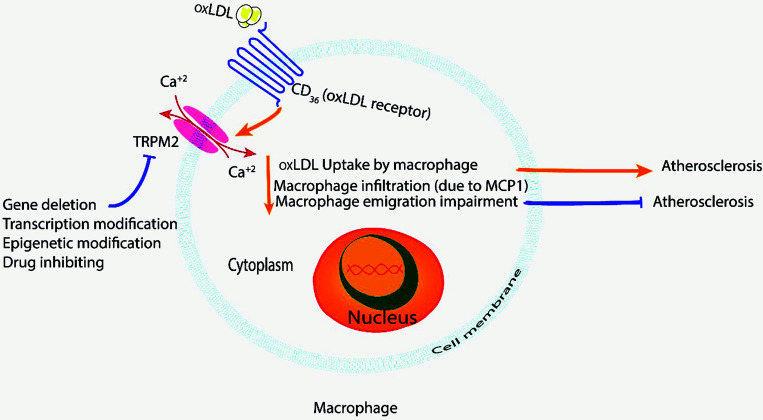
Role of the TRPM2 and CD36 in the atherogenesis process. Inhibiting CD36 reduces TRPM2 signaling activity and furthers atherosclerosis formation. The opposite occurs when inhibiting the TRPM2 downregulation in the activity of the CD36 experience. Abbreviations: oxLDL; oxidative low-density lipoprotein.

Moreover, disruption in the cell membrane permeability regulation in terms of the electrolytes influx/efflux is associated with the upregulation of the NLRP3 activation, caspase-1 activation, and the associated signaling pathway that terminates with intrinsic apoptosis activation and programmed cell death in favorable options [21]. CD36 has been observed to be associated with the activation of the NLRP3 and induce atherosclerosis formation [22]. A recent *in vivo* study demonstrated that deleting Trpm2 or inhibiting TRPM2 activity in cultured macrophages suppressed the CD36 signaling cascade induced by oxidative low-density lipoprotein and TSP1, suggesting that TRPM2 is an effective therapeutic target for atherosclerosis (Fig. **1**) [23].

## 3 LIPIDOME CHANGES IN CORONARY ARTERY DISEASE

Theoretically, changes in lipidome levels are associated with changes in the inflammasome level. However, specific lipid components reduce inflammation biomarkers, and other lipid components elevate the inflammation biomarkers. The elevation of high-density lipoprotein is directly related to the reduction in the levels of interleukin‐1β (IL‐1β), which is dramatically associated with atherosclerosis formation [2].

A recent study demonstrated that patients with cardiovascular disease experience up-regulation in the level of phospholipids and fatty acids in the structure of platelets. Dysregulation of the platelet’s lipid profile is associated with function disturbance and further coagulopathy, suggesting that patients with cardiovascular events require constant and regular administration of antiaggregants. Furthermore, patients with platelet lipid components have a higher incidence rate of stroke and myocardial infarction (MI) [24].

Lipid peroxidation and further sequela are the sources of lipidome changes in patients with coronary artery disease. Acute coronary artery disease is characterized by changes in the lipidome components that vary from the chronic coronary artery disease (stable coronary artery disease). Therefore, the current paper hypothesizes that stable coronary artery disease is characterized by elevation in the plasmalogen lipids, lysophosphatidylethanolamine (LPE), and phosphatidylethanolamine (PE), and reduction in the free stearic acid and fatty acyl esters of hydroxy fatty acids (FAHFA) [25]. At the same time, there is a reduction in the level of malondialdehyde, a marker of oxidative stress [25].

According to recent findings, the changes in the lipidome profile are long-term after the ischemic development. The elevation in the LPE and PE are predominant in the plasma of patients who experience ischemic heart disease [26]. Additionally, an elevation in some fatty acids has been observed during the follow up of the patients with ischemic heart disease. Furthermore, after an ischemic heart attack, a reduction in the level of oxidative stress with time has been observed [25].

A recent prospective, analytical single-center study on a human sample demonstrated that individuals with a history of recent acute coronary syndrome (5 days after the acute coronary syndrome the blood sample was collected) experienced an elevation in the plasmalogen lipids, LPE, and PE. Interestingly, after further follow-up of the patients for a longer period by repeating the blood sampling, a reduction in the levels of the classical biomarkers of lipid peroxidation, including malondialdehyde (MDA), was observed [25]. According to Marzoog *et al*., phosphatidylethanolamine comprises approximately 20% of the total phospholipids of the human myocardiocyte membrane [26]. Therefore, the elevated levels of phosphatidylethanolamine in the plasma of patients with a recent history of acute coronary syndrome are due to myocardiocyte lysis and degradation.

Platelet membrane lipid profile composition is altered in patients with coronary artery disease. Recent findings suggested that perturbance in the ratio of the lipid composition of the platelets is associated with an increased risk of cardiovascular events [24]. Therefore, preserving platelet homeostasis is critical, and prescribing medications that may alter the composition of the platelet lipidome can have bad side effects in terms of increasing the risk of cardiovascular disease development. Thus, the medications, such as antiaggregants and anticoagulants, should be revised.

**Fig. (2):**
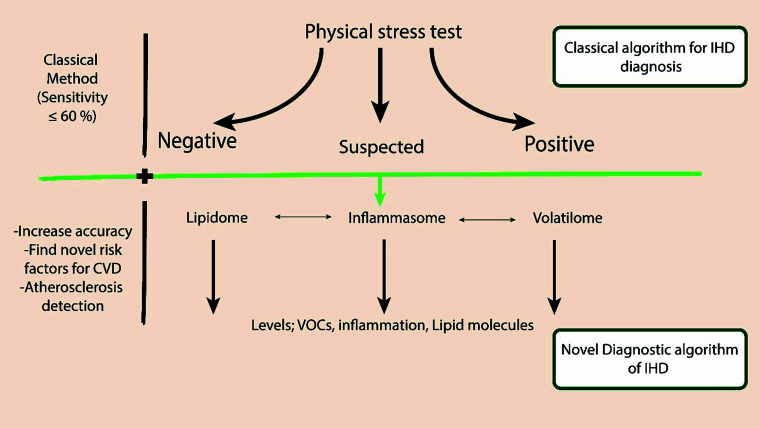
The suggested algorithm includes adding exhaled breath analysis, lipidome, and inflammasome analysis to the classical physical stress test to increase the accuracy of diagnosis. Additionally, using the results can be a novel non-classical risk factor for the prediction of cardiovascular disease. The machine learning model can be added to interpret the results of the lipidome, inflammasome, and volatilome for rapid and worldwide applied method techniques such as programs for global use.

Lipidome and inflammasome changes can be used as a novel therapeutic and diagnostic strategy in coronary artery disease. Moreover, the development of a systemized algorithm in terms of levels of lipidome and inflammasome and the development of a correlation with the VOCs of the exhaled breath are necessary (Fig. **2**).

## 4 IMPLICATIONS OF THE CHANGES IN THE LIPIDOME AND INFLAMMASOME IN THE ANALYSIS OF THE VOCS

The potential changes in the VOCs in patients with CAD remain a challenge for the scientific community. Furthermore, determining the origin of the organic molecules in the exhaled breath is a tough process that requires further investigation. Additionally, the pathways in which these molecules are transported from the damaged myocardiocyte due to CAD to the lungs are released in trace amounts in the exhaled breath analysis. Moreover, the exact molecules that are released from ischemic myocardiocytes into the coronary venous circulation are not obligatory, the same as the molecules released into the exhaled air, since other factors can affect the released molecule, including the reactions that can occur with the released molecules in the bloodstream (organic components of the blood, endothelial cell and it is released molecules) and in the lungs (outdoor breathing air components). The study design should include a control without CAD confirmed by computer cosmography with perfusion of the myocardiocyte, and the second group includes patients with stable CAD I-IV stage. Excluding criteria must have all other diseases (presence of signs of the acute coronary syndrome (myocardial infarction in the last two days) and unstable angina, active infectious and non-infectious inflammatory diseases in the acute phase, respiratory diseases (bronchial asthma, chronic bronchitis, cystic fibrosis), left coronary artery stenosis ≥70%, pulmonary embolism or pulmonary infarction, acute pericarditis/myocarditis, aneurysm of the aorta with dyskinesia, critical aortic stenosis with clinical manifestations, acute thrombophlebitis, active oncopathology, decompensated phase of acute heart failure, neurological disorders (Parkinson's disease, multiple sclerosis, acute psychosis, Guillain-Barré syndrome), arrhythmias (atrial fibrillation, ventricular extrasystole, including ventricular flutter and fibrillation, Wolff-Parkinson-White syndrome, sick sinus syndrome, AV block II-III degree), musculoskeletal disorders, and chronic kidney failure. When using CKD-EPI (updated in 2021), GFR is <30 ml/min per 1.73 m^2^ (III-V), diabetes mellitus) except controlled hypertension, which is acceptable [27].

A poorly designed study conducted to determine the alkane level in patients with unstable angina confirmed the stenosis by coronary angiography and did not confirm the presence or absence of ischemia, which is a severe limitation of the study [28]. The changes presented in the study are not obligatory due to unstable angina because coronary angiography can only confirm the stenosis with an approximate percentage. However, the study suggested an elevation in the level of alkane in patients with unstable angina compared to healthy individuals [28] (supplementary material).

## 5 CURRENT CHALLENGES AND FUTURE THERAPEUTIC AND DIAGNOSTIC POTENTIALS OF EXHALED BREATH ANALYSIS IN ISCHEMIC HEART DISEASE

The limitations of using exhaled breath analysis in the diagnosis of IHD include the lack of standardization. There is a lack of standardization in both breath collection and analytical approaches, which can lead to wide variability in results and affect the accuracy of the diagnosis [29]. The analysis of VOCs in exhaled breath can be influenced by extraneous parameters, such as diet and environment, making it challenging to isolate the specific VOCs associated with IHD [29]. The wide variability in results due to the lack of standardization and the influence of extraneous parameters can hinder the reliability of exhaled breath analysis for the diagnosis of IHD [29]. Furthermore, the acceptance of exhaled breath analysis as a diagnostic tool for IHD among physicians may present a barrier to its widespread clinical implementation [29].

The therapeutics and diagnostics potentials of the exhaled breath analysis involve its use as a rapid, noninvasive diagnostic tool. Exhaled breath analysis has the potential to be used as a rapid, noninvasive diagnostic tool for IHD, offering a relatively inexpensive and noninvasive method for detecting and monitoring a variety of diseases, including cardiovascular disease [30, 31].

Despite the current challenges, exhaled breath analysis holds promise for the future as a noninvasive diagnostic tool for IHD, and further research and technological advancements may overcome the current limitations and lead to its widespread clinical use [31].

## 6 CURRENT CLINICAL IMPLICATIONS OF THE EXHALED BREATH ANALYSIS IN ISCHEMIC HEART DISEASE

As mentioned earlier, exhaled breath analysis has the potential to be used as a novel, rapid, noninvasive diagnostic tool to detect IHD. A proof-of-concept study showed that breath analysis has the potential to differentiate patients who had undergone primary percutaneous intervention for acute myocardial infarction from patients with stable CAD with 97% accuracy and patients with stable CAD from patients without heart disease with 81% accuracy [32]. Breath analysis is rapidly evolving as a new frontier in medical testing. However, further research is needed to validate its use as a diagnostic tool for IHD.

Due to the heterogeny of the mass spectrometry techniques used in the detection of various pathologies, the use of exhaled breath analysis in clinical practice remains in the early period of development. So far, exhaled air analysis has been studied in patients suffering from various diseases, including chronic obstructive lung disease, cancer, asthma, lung cancer, diabetes, arthritis, heart failure, gastric cancer, chronic kidney disease, colorectal cancer, hepatocellular carcinoma, malignant pleural mesothelioma, bladder cancer, pancreatic ductal adenocarcinoma, gastro-oesophageal cancer, peritonitis-shock, head and neck squamous cell carcinoma, multiple sclerosis, and Parkinson’s disease [33-72].

Inflammasome and lipidome changes in patients with coronary artery disease are well established. However, changes in the exhaled VOCs are not investigated in terms of the exact VOCs that exist in patients with confirmed ischemic heart disease. The advantage of using exhaled VOCs is that it improves the diagnostic accuracy of classical physical stress tests, such as bicycle ergometry. Additional advantages include avoiding the usage of expensive and invasive methods as well as time-consuming procedures to confirm the ischemic heart disease, such as CTP, or more invasive methods, such as coronary artery angiography with determination of the fractional flow reserve. Moreover, the clinical applications of the exhaled VOCs include their use as a novel biomarker for scoring the risk of developing IHD or death in healthy or IHD individuals in the next few months or even years according to the concentration (or additional specific criteria) of these specific exhaled VOCs [73, 74]. The novel scoring score can be combined with the currently used SCORE2, SCORE2-OP, and smart risk score, and it can make a program applicable for the scientific community to be used widely and confirm its validity.

Therefore, an ongoing clinical trial (NCT06181799) to determine the exact VOCs in patients with IHD was confirmed by post-stress-induced myocardial perfusion defect on the CTP with vasodilator test (Adenosine triphosphate). The currently existing studies are not well established and have more of a descriptive nature than informing the exact VOCs in patients with IHD. However, in other cardiac studies, such as those on heart failure, there are papers published that are of good quality (Supplementary File **1**).

Indeed, the origin of the VOCs in the exhaled breath analysis of patients with ischemic heart disease has not been studied; likewise, the VOCs in IHD have not been studied. Therefore, there are currently just hypotheses suggesting that the origin of these VOCs is due to the lipid peroxidation of the myocardiocyte or metabolic byproducts (or the presence of the gut microbiota in the atherosclerotic plaque directly) of the atherosclerotic plaque in the coronary arteries or gut microbiota dysbiosis (harmful bacteria overgrowth) in patients with IHD. Furthermore, as a result of this hypothesis, these VOCs in IHD patients can be due to the inflammasome and lipidome changes.

## 7 DISCUSSION

Inflammasomes, which are multi-protein complexes involved in the innate immune response, have been found to play a significant role in coronary artery disease (CAD) [7, 75, 76]. Macrophages, the major cells mediating the inflammatory response, and inflammasomes are both implicated in the development and progression of CAD [75, 77]. The NLRP3 inflammasome, in particular, has been identified as a key contributor to the pathogenesis of angina pectoris, a symptom of CAD [76].

Inflammasome activation leads to the release of pro-inflammatory cytokines, such as interleukin-1β (IL-1β), which contribute to the inflammatory response in CAD [78]. Furthermore, studies suggest that inflammasome activation in CAD may be influenced by factors, such as defective cholesterol efflux, clonal hematopoiesis, and diabetes [78].

Inhibition of inflammasomes or IL-1β has shown promise as a therapeutic target in cardiovascular diseases, including CAD [7,78,79]. Overall, inflammasome changes are implicated in the pathogenesis of CAD and may serve as potential targets for therapeutic interventions [7, 75, 76, 79].

Unfortunately, the molecular biopathophysiology that stands behind the changes in the lipidome, inflammasome, and volatilome is not well established and requires further elaboration. Probably, changes in the lipidome and inflammasome are reflected in the breathome, and there are no constant changes in exhaled breath volatiome composition. The primary changes start with the changes in the morphology of the vascular structure, including atherosclerosis formation due to the dysregulation of the lipidome and inflammasome level due to the predominance of the aggression system on the protection system on the cellular and subcellular level [8, 11-16, 26, 80-89]. Thereafter, due to the occlusion of the elastic and musculo-elastic arteries by atherosclerosis, a disturbance in the stream of blood in the vasculature leads to further endothelial damage. The occlusion of the bloodstream to the organs, such as the heart, results in ischemic development. In terms of prolonged ischemia, a necrosis stage develops, where the intracellular metabolic components are released into the circulation.

Furthermore, the early stages of ischemia do not have the changes in these biomarkers. However, individuals suffer from clinically silent ischemia, and its detection in the early period remains a challenge, whereas classical physical stress tests can detect it in 60% of cases. These limitations in the currently used methods are not sufficient for early diagnosis of latent ischemic heart disease, and developing a surrogate method is urgent.

## CONCLUSION

In summary, while there are challenges, such as standardization, the influence of extraneous parameters, and physician acceptance, exhaled breath analysis holds significant promise as a rapid, noninvasive diagnostic tool for IHD and is considered a novel frontier in medical testing, with the potential for future applications in the diagnosis and monitoring of cardiovascular diseases, including IHD.

Lipidome and inflammasome changes can be used as a novel therapeutic and diagnostic strategy in coronary artery disease [90-92]. The combination of the exhaled breath analysis with the lipidome and the inflammasome levels holds a promising future for effectively and safely detecting ischemic heart disease with high efficacy and safety. Moreover, the implications of artificial learning models can dramatically improve the accuracy of prediction for morbidity and mortality by using a combination of the lipidome, inflammasome, and volatilome compositions. Additionally, the changes in these biomarkers can be used as novel non-classical risk factors for the development of ischemic heart disease. Hence, we suggest the development of a new method for CVD risk evaluation by adding inflammasome, lipidome, and volatilome to the classical SCORE and SCORE2-OP to improve the accuracy. This hypothesis is under development by Marzoog and co-authors to develop a program that includes all these parameters with special formulations.

Moreover, changes in the lipidome, inflammasome, and exhaled breath analysis can be used to enhance the diagnostic accuracy of classical physical stress tests, such as bicycle and treadmill ergometry. Additionally, the detection of atherosclerosis is possible through the usage of these changes due to the fact that most of the incidences of ischemic heart disease pathophysiology include the occlusion of the coronary arteries by atherosclerosis [93]. However, a challenging issue is confirming that the changes in these biomarkers (inflammasome, lipidome, and volatilome) are associated with atherosclerosis of the coronary arteries or other arteries, such as brachiocephalic or carotid arteries, which are the most common sites for atherosclerosis.

## LIST OF ABBREVIATIONS

CAD: Coronary Artery Disease
FAHFA: Free Stearic Acid and Fatty Acyl Esters of Hydroxy Fatty Acids
IHD: Ischemic Heart Disease
IL‐1β: Interleukin‐1β
LPE: Lysophosphatidylethanolamine
MDA: Malondialdehyde
MI: Myocardial Infarction
PE: Phosphatidylethanolamine
TLR9: Toll-like Receptor 9
VOCs: Volatile Organic Compounds

## References

[1] Tsao C.W., Aday A.W., Almarzooq Z.I. (2023). Heart disease and stroke statistics—2023 update: A report from the American heart association.. Circulation.

[2] Salonen I., Huttunen K., Hirvonen M.R. (2012). Exhaled nitric oxide and atherosclerosis.. Eur. J. Clin. Invest..

[3] Nurmohamed N.S., Kraaijenhof J.M., Mayr M. (2023). Proteomics and lipidomics in atherosclerotic cardiovascular disease risk prediction.. Eur. Heart J..

[4] Močnik M., Marčun Varda N. (2023). Lipid biomarkers and atherosclerosis—old and new in cardiovascular risk in childhood.. Int. J. Mol. Sci..

[5] Zhu D., Vernon S.T., D’Agostino Z. (2023). Lipidomics profiling and risk of coronary artery disease in the BioHEART-CT discovery cohort.. Biomolecules.

[6] Dugani S.B., Moorthy M.V., Li C. (2021). Association of lipid, inflammatory, and metabolic biomarkers with age at onset for incident coronary heart disease in women.. JAMA Cardiol..

[7] Olsen M.B., Gregersen I., Sandanger Ø. (2022). Targeting the inflammasome in cardiovascular disease.. JACC Basic Transl. Sci..

[8] Abdullah Marzoog B. (2024). Cytokines and regulating epithelial cell division.. Curr. Drug Targets.

[9] Thacker S.G., Zarzour A., Chen Y. (2016). High‐density lipoprotein reduces inflammation from cholesterol crystals by inhibiting inflammasome activation.. Immunology.

[10] Ridker P.M., Everett B.M., Thuren T. (2017). Antiinflammatory therapy with canakinumab for atherosclerotic disease.. N. Engl. J. Med..

[11] Marzoog B.A. (2023). Tree of life: Endothelial cell in norm and disease, the good guy is a partner in crime!. Anat. Cell Biol..

[12] Marzoog B.A. (2024). Endothelial dysfunction under the scope of arterial hypertension, coronary heart disease, and diabetes mellitus using the angioscan.. Cardiovasc. Hematol. Agents Med. Chem..

[13] Marzoog B.A. (2023). Autophagy behavior in post-myocardial infarction injury.. Cardiovasc. Hematol. Disord. Drug Targets.

[14] Marzoog B. (2022). Lipid behavior in metabolic syndrome pathophysiology.. Curr. Diabetes Rev..

[15] Marzoog B.A. (2023). The metabolic syndrome puzzles; Possible pathogenesis and management.. Curr. Diabetes Rev..

[16] Marzoog B.A. (2023). Endothelial cell autophagy in the context of disease development.. Anat. Cell Biol..

[17] Abdullah Marzoog B. (2023). Adaptive and compensatory mechanisms of the cardiovascular system and disease risk factors in young males and females.. Emir. Med. J..

[18] Xie D., Guo H., Li M. (2023). Splenic monocytes mediate inflammatory response and exacerbate myocardial ischemia/reperfusion injury in a mitochondrial cell-free DNA-TLR9-NLRP3-dependent fashion.. Basic Res. Cardiol..

[19] Duewell P., Kono H., Rayner K.J. (2010). NLRP3 inflammasomes are required for atherogenesis and activated by cholesterol crystals.. Nature.

[20] Kawaguchi M., Takahashi M., Hata T. (2011). Inflammasome activation of cardiac fibroblasts is essential for myocardial ischemia/reperfusion injury.. Circulation.

[21] Muñoz-Planillo R., Kuffa P., Martínez-Colón G., Smith B.L., Rajendiran T.M., Núñez G. (2013). K^+^ efflux is the common trigger of NLRP3 inflam-masome activation by bacterial toxins and particulate matter.. Immunity.

[22] Sheedy F.J., Grebe A., Rayner K.J. (2013). CD36 coordinates NLRP3 inflammasome activation by facilitating intracellular nucleation of soluble ligands into particulate ligands in sterile inflammation.. Nat. Immunol..

[23] Zong P., Feng J., Yue Z. (2022). TRPM2 deficiency in mice protects against atherosclerosis by inhibiting TRPM2–CD36 inflammatory axis in macrophages.. Nature Cardiovascular Research..

[24] Harm T., Dittrich K., Brun A. (2023). Large-scale lipidomics profiling reveals characteristic lipid signatures associated with an increased cardiovascular risk.. Clin. Res. Cardiol..

[25] Kosek V., Hajšl M., Bechyňská K. (2022). Long-term effects on the lipidome of acute coronary syndrome patients.. Metab.

[26] Marzoog B.A., Vlasova T.I. (2021). Membrane lipids under norm and pathology.. Eur J Clin Exp Med.

[27] Gibbons R.J., Balady G.J., Beasley J.W. (1997). ACC/AHA guidelines for exercise testing: Executive summary.. Circulation.

[28] Phillips M., Cataneo R.N., Greenberg J., Grodman R., Salazar M. (2003). Breath markers of oxidative stress in patients with unstable angina.. Heart Dis..

[29] Das S., Pal S., Mitra M. (2016). Significance of exhaled breath test in clinical diagnosis: A special focus on the detection of diabetes mellitus.. J. Med. Biol. Eng..

[30] Cikach F.S., Dweik R.A. (2012). Cardiovascular biomarkers in exhaled breath.. Prog. Cardiovasc. Dis..

[31] Sharma A., Kumar R., Varadwaj P. (2023). Smelling the disease: Diagnostic potential of breath analysis.. Mol. Diagnosis. Ther..

[32] Nardi Agmon I., Broza Y.Y., Alaa G. (2022). Detecting coronary artery disease using exhaled breath analysis.. Cardiology.

[33] Trefz P., Obermeier J., Lehbrink R., Schubert J.K., Miekisch W., Fischer D.C. (2019). Exhaled volatile substances in children suffering from type 1 diabetes mellitus: Results from a cross-sectional study.. Sci. Rep..

[34] van de Kant K.D.G., van der Sande L.J.T.M., Jöbsis Q., van Schayck O.C.P., Dompeling E. (2012). Clinical use of exhaled volatile organic compounds in pulmonary diseases: A systematic review.. Respir. Res..

[35] Amal H., Leja M., Funka K. (2016). Breath testing as potential colorectal cancer screening tool.. Int. J. Cancer.

[36] Chapman E.A., Baker J., Aggarwal P. (2023). GC-MS techniques investigating potential biomarkers of dying in the last weeks with lung cancer.. Int. J. Mol. Sci..

[37] Chung J., Akter S., Han S. (2022). Diagnosis by volatile organic compounds in exhaled breath from patients with gastric and colorectal cancers.. Int. J. Mol. Sci..

[38] Sukaram T., Tansawat R., Apiparakoon T. (2022). Exhaled volatile organic compounds for diagnosis of hepatocellular carcinoma.. Sci. Rep..

[39] Politi L., Monasta L., Rigressi M.N. (2021). Discriminant profiles of volatile compounds in the alveolar air of patients with squamous cell lung cancer, lung adenocarcinoma or colon cancer.. Molecules.

[40] Di Gilio A., Catino A., Lombardi A. (2020). Breath analysis for early detection of malignant pleural mesothelioma: Volatile organic compounds (VOCs) determination and possible biochemical pathways.. Cancers.

[41] Catino A., de Gennaro G., Di Gilio A. (2019). Breath analysis: A systematic review of volatile organic compounds (VOCs) in diagnostic and therapeutic management of pleural mesothelioma.. Cancers.

[42] Rodrigues D., Pinto J., Araújo A.M. (2018). Volatile metabolomic signature of bladder cancer cell lines based on gas chromatography–mass spectrometry.. Metabolomics.

[43] Princivalle A., Monasta L., Butturini G., Bassi C., Perbellini L. (2018). Pancreatic ductal adenocarcinoma can be detected by analysis of volatile organic compounds (VOCs) in alveolar air.. BMC Cancer.

[44] Chin S.T., Romano A., Doran S.L.F., Hanna G.B. (2018). Cross-platform mass spectrometry annotation in breathomics of oesophageal-gastric cancer.. Sci. Rep..

[45] Brekelmans M.P., Fens N., Brinkman P. (2016). Smelling the diagnosis: The electronic nose as diagnostic tool in inflammatory arthritis. A case-reference study.. PLoS One.

[46] DeLano F.A., Chow J., Schmid-Schönbein G.W. (2017). Volatile decay products in breath during peritonitis shock are attenuated by enteral blockade of pancreatic digestive proteases.. Shock.

[47] Krilaviciute A., Heiss J.A., Leja M., Kupcinskas J., Haick H., Brenner H. (2015). Detection of cancer through exhaled breath: A systematic review.. Oncotarget.

[48] Hanna G.B., Boshier P.R., Markar S.R., Romano A. (2019). Accuracy and methodologic challenges of volatile organic compound–based exhaled breath tests for cancer diagnosis.. JAMA Oncol..

[49] Gruber M., Tisch U., Jeries R. (2014). Analysis of exhaled breath for diagnosing head and neck squamous cell carcinoma: A feasibility study.. Br. J. Cancer.

[50] Bajtarevic A., Ager C., Pienz M. (2009). Noninvasive detection of lung cancer by analysis of exhaled breath.. BMC Cancer.

[51] Xu Z., Broza Y.Y., Ionsecu R. (2013). A nanomaterial-based breath test for distinguishing gastric cancer from benign gastric conditions.. Br. J. Cancer.

[52] Peled N., Hakim M., Bunn P.A. (2012). Non-invasive breath analysis of pulmonary nodules.. J. Thorac. Oncol..

[53] Ionescu R., Broza Y., Shaltieli H. (2011). Detection of multiple sclerosis from exhaled breath using bilayers of polycyclic aromatic hydrocarbons and single-wall carbon nanotubes.. ACS Chem. Neurosci..

[54] Buszewski B., Ligor T., Jezierski T., Wenda-Piesik A., Walczak M., Rudnicka J. (2012). Identification of volatile lung cancer markers by gas chromatographymass spectrometry: Comparison with discrimination by canines.. Anal. Bioanal. Chem..

[55] Stott S., Broza Y.Y., Gharra A., Wang Z., Barker R.A., Haick H. (2022). The utility of breath analysis in the diagnosis and staging of parkinson’s disease.. J. Parkinsons Dis..

[56] Marcondes-Braga F.G., Gioli-Pereira L., Bernardez-Pereira S. (2020). Exhaled breath acetone for predicting cardiac and overall mortality in chronic heart failure patients.. ESC Heart Fail..

[57] Marcondes-Braga F.G., Batista G.L., Bacal F., Gutz I. (2016). Exhaled breath analysis in heart failure.. Curr. Heart Fail. Rep..

[58] Bykova A.A., Malinovskaya L.K., Chomakhidze P.S. (2019). Exhaled breath analysis in diagnostics of cardiovascular diseases.. Kardiologiia.

[59] Bykova AA, Malinovskaya LK, Trushina OV (2019). Exhaled breath analysis in diagnosis of chronic heart failure with reduced left ventricular ejection fraction. Cardiology and cardiovascular surgery.

[60] Marcondes-Braga F.G., Batista G.L., Gutz I.G.R. (2016). Impact of exhaled breath acetone in the prognosis of patients with heart failure with reduced ejection fraction (HFrEF).. PLoS One.

[61] Malinovskaya LK, Bykova AA, Chomahidze PSH, Kopylov PHYU, Syrkin AL, Betelin VB (2018). P3758Exhaled breath analysis in the differen-tial diagnostics of heart failure.. . Eur Heart J.

[62] Biagini D., Lomonaco T., Ghimenti S. (2017). Determination of volatile organic compounds in exhaled breath of heart failure patients by needle trap micro-extraction coupled with gas chromatographytandem mass spectrometry.. J. Breath Res..

[63] Yokokawa T., Sato T., Suzuki S. (2017). Elevated exhaled acetone concentration in stage C heart failure patients with diabetes mellitus.. BMC Cardiovasc. Disord..

[64] Yokokawa T., Sato T., Suzuki S. (2018). Change of exhaled acetone concentration levels in patients with acute decompensated heart failure a preliminary study.. Int. Heart J..

[65] Zhou Q., Wang Q., Chen B. (2019). Factors influencing breath analysis results in patients with diabetes mellitus.. J. Breath Res..

[66] Broza Y.Y., Khatib S., Gharra A. (2019). Screening for gastric cancer using exhaled breath samples.. Br. J. Surg..

[67] Peled N., Fuchs V., Kestenbaum E.H., Oscar E., Bitran R. (2021). An update on the use of exhaled breath analysis for the early detection of lung cancer.. Lung Cancer.

[68] Wang M.H., Yuk-Fai Lau S., Chong K.C. (2017). Estimation of clinical parameters of chronic kidney disease by exhaled breath full-scan mass spectrometry data and iterative PCA with intensity screening algorithm.. J. Breath Res..

[69] Zeng Q., Li P., Cai Y. (2016). Detection of creatinine in exhaled breath of humans with chronic kidney disease by extractive electrospray ionization mass spectrometry.. J. Breath Res..

[70] Badjagbo K. (2012). Exhaled breath analysis for early cancer detection: principle and progress in direct mass spectrometry techniques.. Clinical Chemistry and Laboratory Medicine (CCLM).

[71] Chan M.J., Li Y.J., Wu C.C. (2020). Breath ammonia is a useful biomarker predicting kidney function in chronic kidney disease patients.. Biomedicines.

[72] Rodríguez-Aguilar M., Ramírez-García S., Ilizaliturri-Hernández C. (2019). Ultrafast gas chromatography coupled to electronic nose to identify volatile biomarkers in exhaled breath from chronic obstructive pulmonary disease patients: A pilot study.. Biomed. Chromatogr..

[73] Marzoog B.A., Volatilome A. (2024). Volatilome: A novel tool for risk scoring in ischemic heart disease.. Curr. Cardiol. Rev..

[74] Marzoog B. (2024). Breathomics detect the cardiovascular disease: Delusion or dilution of the metabolomic signature.. Curr. Cardiol. Rev..

[75] Zhang Y., Tu J., Li Y. (2023). Inflammation macrophages contribute to cardiac homeostasis.. Cardiology. Plus.

[76] Sharma I., Behl T., Bungau S. (2021). Understanding the role of inflammasome in angina pectoris.. Curr. Protein Pept. Sci..

[77] Lavine K.J., Epelman S., Uchida K. (2014). Distinct macrophage lineages contribute to disparate patterns of cardiac recovery and remodeling in the neonatal and adult heart.. Proc. Natl. Acad. Sci. USA.

[78] Rajamäki K., Mäyränpää M.I., Risco A. (2016). p38δ MAPK.. Arterioscler. Thromb. Vasc. Biol..

[79] Tall A.R., Bornfeldt K.E. (2023). Inflammasomes and atherosclerosis: A mixed picture.. Circ. Res..

[80] Marzoog B.A. (2023). Autophagy behavior in endothelial cell dysfunction.. Emir. Med. J..

[81] Marzoog B.A. (2024). Nicotinamide mononucleotide in the context of myocardiocyte longevity.. Curr. Aging Sci..

[82] Marzoog B.A. (2023). Transcription factors – The essence of heart regeneration: A potential novel therapeutic strategy.. Curr. Mol. Med..

[83] Abdullah Marzoog B. (2023). Caveolae’s behavior in norm and pathology.. Emir. Med. J..

[84] Abdullah Marzoog B. (2023). Cell physiological behavior in the context of local hypothermia.. Emir. Med. J..

[85] Marzoog B.A. (2023). Autophagy behavior in endothelial cell regeneration.. Curr. Aging Sci..

[86] Abdullah Marzoog B. (2023). Autophagy behavior under local hypothermia in myocardiocytes injury.. Cardiovasc. Hematol. Agents Med. Chem..

[87] Marzoog B.A. (2024). Incidence rate of post coronary artery shunt complications; Age dependent!. Cardiovasc. Hematol. Agents Med. Chem..

[88] Marzoog B.A. (2024). Endothelial cell aging and autophagy dysregulation.. Cardiovasc. Hematol. Agents Med. Chem..

[89] Marzoog B.A., Vlasova T.I. (2022). Myocardiocyte autophagy in the context of myocardiocytes regeneration: A potential novel therapeutic strategy.. Egypt. J. Med. Hum. Genet..

[90] Tabassum R., Ripatti S. (2021). Integrating lipidomics and genomics: Emerging tools to understand cardiovascular diseases.. Cell. Mol. Life Sci..

[91] Abrahams T., Nicholls S.J. (2023). Perspectives on the success of plasma lipidomics in cardiovascular drug discovery and future challenges.. Expert Opin. Drug Discov..

[92] Hinterwirth H., Stegemann C., Mayr M. (2014). Lipidomics.. Circ. Cardiovasc. Genet..

[93] Severino P., D’Amato A., Pucci M. (2020). Ischemic heart disease pathophysiology paradigms overview: From plaque activation to microvascular dysfunction.. Int. J. Mol. Sci..

